# Dr. M. Sudhakar Shetty

**Published:** 2011

**Authors:** Shantharam Shetty

**Affiliations:** *Past President of Indian Orthopaedic Association, Vice Chancellor, Nitte University, Chairman, Tejasvini Hospital, Mangalore, India. E-mail: shettyortho@hotmail.com*

**Figure:**
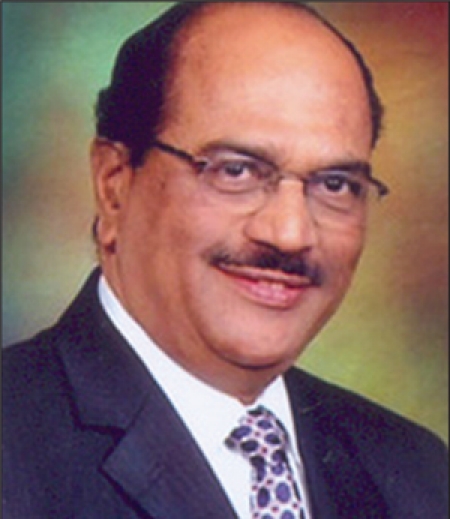
Dr. Mundkur Sudhakar Shetty (1946-2011)

Mundkur Sudhakar Shetty was born in an illustrious Bunt family on 04-12-1946. He graduated from the prestigious Government Medical College, Bangalore and earned his M.S (Orth) degree from Bombay University. Working as a resident in Seth G.S Medical College, Bombay, one of the busiest orthopedic centers of the country, he had acquired abundant skills in orthopedic surgery and with a will and vision to serve his own people of his home district, he came and settled down in Mangalore and joined the Kasturba Medical College as Asst. Professor in 1975. Always determined to better himself, in 1980, he proceeded to Liverpool, worked with George Bentley and he passed M.Ch (Orth) with distinction.

He served Kasturba Medical College with distinction for more than 32 years. He was the Professor and head of the Department of Orthopedics for a full term. After retirement, he joined Yenepoya Medical College as a Professor and rose to be the Vice Dean of this Medical College.

A rare blend of a perfect clinician, excellent teacher and a brilliant surgeon, Dr. Sudhakar had trained more than 200 postgraduate students who are spread all round the globe. He had an inherent thirst for teaching; he was instrumental in starting the popular Mangalore Orthopedic Course which used to draw student delegates from all round the country. A visionary that he was, he started the Canara Orthopaedic Society which today has more than 300 members imparting fellowship, understanding and knowledge treasure to its members. He was the past president of Karnataka Orthopaedic Association and Orthopaedic Association of South Indian States as well.

A very active member of the Indian Orthopaedic Association he was awarded the coveted Honorary Membership and Life time Achievement Award which he richly deserved.

A keen badminton player and a social worker of repute, Dr. Shetty was known for his compassion to his patients. People like Sudhakar can never die. They leave behind their footsteps in the sands of time for people to remember, patients and students to thank eternally.

He was my colleague for more than 30 years. We will miss him, his scholarly lectures, his collective wisdom, more than all his wit and ever-ready helping hand. A perfect family man Sudhakar is survived by his wife Amrithakala, a great hostess, and two lovely daughters Anupa and Ashika and thousands of admirers.

May his soul rest in eternal peace.

